# Potential Activities of Freshwater Exo- and Endo-Acting Extracellular Peptidases in East Tennessee and the Pocono Mountains

**DOI:** 10.3389/fmicb.2018.00368

**Published:** 2018-03-06

**Authors:** Lauren Mullen, Malcolm X Shabazz High School Aquatic Biogeochemistry Team, Kim Boerrigter, Nicholas Ferriero, Jeff Rosalsky, Abigail van Buren Barrett, Patrick J. Murray, Andrew D. Steen

**Affiliations:** ^1^Department of Earth and Planetary Sciences, University of Tennessee, Knoxville, Knoxville, TN, United States; ^2^Malcolm X Shabazz High School, Newark, NJ, United States; ^3^Harvard College, Cambridge, MA, United States; ^4^Pocono Environmental Education Center, Dingmans Ferry, PA, United States

**Keywords:** extracellular enzymes, aminopeptidase, endopeptidase, freshwater, trypsin, protein

## Abstract

Proteins constitute a particularly bioavailable subset of organic carbon and nitrogen in aquatic environments but must be hydrolyzed by extracellular enzymes prior to being metabolized by microorganisms. Activities of extracellular peptidases (protein-degrading enzymes) have frequently been assayed in freshwater systems, but such studies have been limited to substrates for a single enzyme [leucyl aminopeptidase (Leu-AP)] out of more than 300 biochemically recognized peptidases. Here, we report kinetic measurements of extracellular hydrolysis of five substrates in 28 freshwater bodies in the Delaware Water Gap National Recreation Area in the Pocono Mountains (PA, United States) and near Knoxville (TN, United States), between 2013 and 2016. The assays putatively test for four aminopeptidases (arginyl aminopeptidase, glyclyl aminopeptidase, Leu-AP, and pyroglutamyl aminopeptidase), which cleave *N*-terminal amino acids from proteins, and trypsin, an endopeptidase, which cleaves proteins mid-chain. Aminopeptidase and the trypsin-like activity were observed in all water bodies, indicating that a diverse set of peptidases is typical in freshwater. However, ratios of peptidase activities were variable among sites: aminopeptidases dominated at some sites and trypsin-like activity at others. At a given site, the ratios remained fairly consistent over time, indicating that they are driven by ecological factors. Studies in which only Leu-AP activity is measured may underestimate the total peptidolytic capacity of an environment, due to the variable contribution of endopeptidases.

## Introduction

Kinetics of extracellular enzymes can give insight into the rates and pathways of organic matter processing in the environment (Schimel and Weintraub, 2003; Arnosti et al., 2014; Sinsabaugh et al., 2014). Diverse classes of extracellular enzymes have been observed in freshwaters, including peptidases, polysaccharide hydrolases, phosphatases, lipases, peroxidases, and laccases (Findlay and Sinsabaugh, 1999). Peptidases can be particularly valuable to microbial communities, because proteins provide organic N as well as C, and because protein-like organic matter is on average more bioavailable than bulk natural organic matter (Fellman et al., 2010). The peptidases are a highly diverse class of enzymes: more than 300 distinct peptidases have been identified by function (McDonald et al., 2009) and many more have been identified by structure (Rawlings et al., 2016). Nevertheless, environmental studies have focused almost exclusively on the activity of a single extracellular peptidase, leucyl aminopeptidase (Leu-AP), which preferentially cleaves leucine from the *N*-terminus of proteins or peptides (Arnosti et al., 2014). In seawater, a diverse suite of aminopeptidases and endopeptidases (which cleave peptide bonds within proteins; Hooper, 2002), from bacteria as well as protists, are required for complete breakdown of proteins (Thao et al., 2014). Ratios of LeuAP and other aminopeptidases to endopeptidases can vary widely (Obayashi and Suzuki, 2005, 2008; Steen and Arnosti, 2013). The extent of this variation in freshwaters, and therefore the extent to which potential LeuAP activities represent total peptidolytic potential of freshwater ecosystems, remains unknown.

In order to constrain the degree to which LeuAP activities represent the total range of extracellular peptidases active in freshwaters, we assayed the potential activities of five different classes of extracellular peptidases in 28 freshwater bodies in southwest Pennsylvania (PA) and east Tennessee (TN) between 2013 and 2016. In addition to Leu-AP, we used substrates that putitively assay arginyl aminopeptidase (ArgAP), prolyl aminopeptidase, glycyl aminopeptidase (GlyAP), pyroglutamyl aminopeptidase, and trypsin. The first four of these are aminopeptidases while trypsin is an endopeptidase. Uniquely among amino acids in the substrates assayed here, pyroglutamic acid is not directly encoded by DNA and is not typically abundant in biomass. However, it does exist in low quantities in some environmentally relevant biomolecules, for instance, bacteriorhodopsin (Gerber et al., 1979). Freshwater organic matter contains a complex mixture of proteins and protein-like molecules that require a diverse suite of extracellular enzymes to efficiently remineralize (Arnosti et al., 2014). A better understanding of the nature of extracellular peptidases in aquatic environments could therefore shed light on the mechanisms by which organic matter is oxidized in such systems.

## Materials and Methods

### Sites and Sample Collection

**FIGURE 1:**
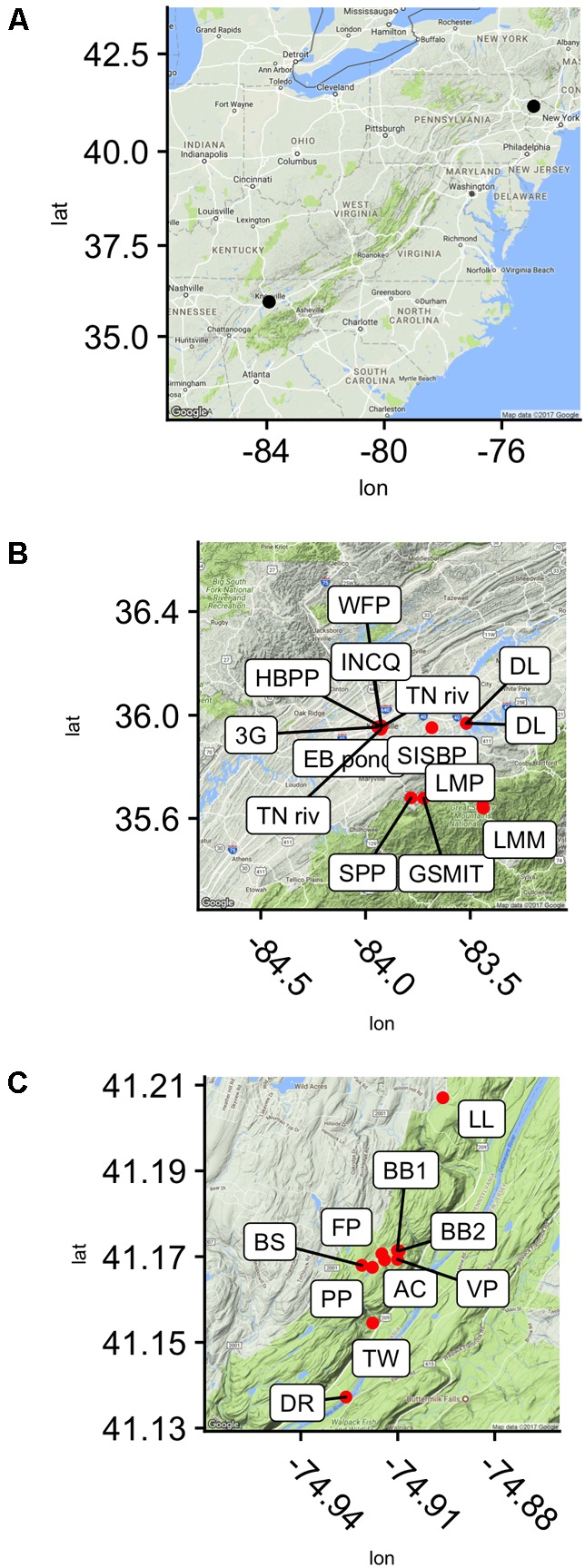
Sampling sites in reference to the east coast of the United states **(A)** and seen in higher resolution, in the Delaware Water Gap National Recreation Area, Dingmans Ferry, PA, United States **(C)** and near Knoxville, TN, United States **(B)**.

Samples were collected from 28 locations in and around Knoxville, TN, United States, and in the Pocono Mountains of eastern PA near the Pocono Environmental Education Center in 2013, 2015, and 2016 (PEEC; **Figure 1** and **Table 1**). Water samples were collected by hand in acid-rinsed, 1-L polyethylene bottles. *In situ* water temperature was measured at the time of sampling. For the Knoxville samples, pH was measured by electronic pH meter (Accumet AB150) upon return to the University of TN. For the PEEC samples, pH was measured using pH strips (2013–2015, 2016 YSI Pro DSS Sonde). A portion of the collected samples have missing pH values; these samples have been recorded as n.m in **Table 1**. Methods were developed through progressive years of the study and evolved to be more efficient; pH data were collected for most samples but were neglected in the early studies. Samples were kept at *in situ* temperature in the dark and returned to the lab within 1 h for enzyme assays. To assess temporal variability in peptidase activities, a short time series of six samples each was collected from the Third Creek (3rd) and Volunteer Landing (VL) sites during the period from June 8 to July 6, 2015.

**Table 1:** Selected sampling sites and their corresponding GPS coordinates, temperature, pH, date, and time.

Site initials	Site name	Latitude	Longitude	Temperature	pH	Sample date	Sample time	State
TN	Tennessee River	35.956201	83.920999	30	6.81	5/13/15	13:30	TN
EB	Estabrook Fish Pond	35.955863	83.923597	29	7.88	5/14/15	13:20	TN
GSMIT	Great Smokey Mountain Institute at Tremont	35.678246	83.722413	26	7.11	5/15/15	11:00	TN
SPP	Special People’s Park	35.67984	83.782517	26	7.19	5/15/15	11:20	TN
DL	Douglas Lake	35.969042	83.518601	32	7.43	5/18/15	8:00	TN
HBPP	Hesler Biology Plant Pond	35.956517	83.926578	30	6.86	5/18/15	9:00	TN
DL2	Douglas Lake 2	35.969042	83.518601	30	7.45	5/19/15	8:00	TN
SISBP	Seven Islands State Birding Park	35.951805	83.683438	28	7.38	5/19/15	8:47	TN
3G	Third Creek Greenway	35.954235	83.942073	29	7.55	5/20/15	9:10	TN
TN2	Tennessee River Water Plant	35.945633	83.926757	25	8.16	5/21/15	8:30	TN
WFP	World’s Fair Park	35.958352	83.924428	20	7.66	5/21/15	8:40	TN
ECD	Eastman Chemical Dam	36.511133	82.537032	20	7.81	5/22/15	5:00	TN
INCQ	Ijams Nature Center Quarry	35.958352	83.924428	26	8.04	5/22/15	8:20	TN
BS	Briscoe Swamp	41.16796667	–74.9211	21	n.m	5/28/13	8:55	PA
BS2	Briscoe Swamp	41.16796667	–74.9211	21	8.11	6/01/16	18:20	PA
BW	Birchwood Lakes	41.242638	74.920148	23	n.m	5/28/13	n.m	PA
DR	Delaware River	41.13724	74.926029	25	7.21	5/28/15	12:35	PA
FP	Front Pond	41.170668	–74.91492	25	7	5/27/15	12:10	PA
FP2	Front Pond 2	41.170668	–74.91492	25	7	6/01/16	14:30	PA
LL	Loch Lomond	41.20705	–74.89605	23	7	5/28/15	9:40	PA
PP	Pickerel Pond	41.167504	–74.91786	27	7	5/27/15	14:06	PA
PP2	Pickerel Pond 2	41.167504	–74.91786	27	7.5	6/01/16	13:42	PA
RR	Raritan River	40.511438	–74.30216	27	8.0	5/28/15	11:00	PA
RR2	Raritan River 2	40.511438	–74.30216	27	8.0	6/02/16	10:00	PA
SI	Shark River Inlet	40.187105	–74.00984	25	n.m	5/27/13	n.m	PA
SW	Scenic Waters	41.171494	–74.90971	24	7	5/27/15	n.m	PA
SW2	Scenic Waters 2	41.171494	–74.90971	24	7	6/02/16	15:00	PA
TW	Tumbling Waters Creek	41.15451667	–74.91775	18	n.m	5/28/15	9:16	PA
AC	Alicia’s Creek	41.16923333	–74.91406	20	7	5/27/15	13:58	PA
BB1	Basketball Court Pond 1	41.171401	–74.90943	23	7.12	5/28/15	13:53	PA
BB2	Basketball Court Pond 2	41.171371	–74.90930	23	7	6/02/16	10:38	PA
VP	Vernal Pool	41.16935	74.914066	23	n.m	5/28/13	10:38	PA

*n.m. indicates ‘not measured’.*

### Enzyme Assays

Enzyme assays were performed using fluorogenic substrates (Hoppe, 1983) according to a modified version of the protocol described by Steen and Arnosti (2011). The following substrates were used: Arg-7-aminomethylcoumarin (AMC), Gly-AMC, Leu-AMC, Pyr-AMC, and Z-GlyGlyArg-AMC. Details of substrates are given in **Table 2**.

**Table 2:** Substrates used.

Substrate	Abbreviation	Supplier	Product number
L-Arginine-7-amido-4-methylcoumarin HCl	Arg-AMC	Sigma-Aldrich	A2027
Glycine 7-amido-4-methylcoumarin (Gly-AMC)	Gly-AMC	Bachem	03351
L-Leucine 7-amido-4-methylcoumarin (Leu-AMC)	Leu-AMC	Chem-Impex International	06122
L-Pyroglutamic acid 7-amido-4-methylcoumarin	Pyr-AMC	Biosynth Chemistry and Biology	P-8500
Carboxybenzoyl-glycine-glycine-arginine 7-amido-4-methylcoumarin HCl	Z-GlyGlyArg-AMC	Bachem	I1140.0025

*Suppliers and product numbers are examples. Different suppliers for identical molecules were used over the course of the study.*

The four aminopeptidase substrates were chosen to represent a broad range of amino R group chemistries, including non-polar (Leu), polar (Arg), small (Gly), and pyroglutamic acid, which is non-proteinogenic and which has an unusual cyclic R group. Z-GlyGlyArg-AMC, the only endopeptidase substrate used due to cost constraints, was chosen because hydrolysis of it was consistently observed (Obayashi and Suzuki, 2005, 2008). We note that the Z-(carboxybenzyl-) group on this substrate is a bulky protecting group that prevents sequential hydrolysis of the substrate by aminopeptidases. Throughout this manuscript, we use the Enzyme Commission (EC) system to refer to the enzymes that hydrolyze these substrates, in which enzymes are classified according to their function without regard to structure (Webb, 1992) because we lack any data (e.g., nucleic acid sequences) on enzyme structure. This is a shortcut: multiple peptidases are capable of catalyzing the hydrolysis of each of these substrates, as discussed below. For instance, both trypsin (EC 3.4.21.4) and oligopeptidase B (EC 3.4.21.83) are capable of catalyzing the hydrolysis of peptide bonds with *N*-adjacent Arg, despite major structural differences. The enzyme names used here are therefore consistent with specific enzyme classes, but not necessarily diagnostic of them.

In 2013, saturation curves (measurements of substrate hydrolysis rate as a function of substrate concentration at 0, 50, 100, 150, 200, 250, 300, and 400 μM) were measured at each site, with a single live replicate and matching killed control (boiled for ca. 5 min) at each concentration, plus triplicate live measurements at 400 μM. In 2014–2016, triplicate, saturating concentrations of 400 μM substrate were used in each incubation as well as a single killed control; 40 μL of substrate (10 mM stock concentration, dissolved in 90% MilliQ-H_2_O/10% DMSO) was added to 100 μL of phosphate buffer (100 mM, pH 7.5) and 860 μL unfiltered sample, in a 1-mL methacrylate cuvette. The cuvette was capped and mixed by hand. Measurements were taken approximately every 20 min for 2 h using a Promega Glomax Jr. (Ex 365 nm, Em 410–460 nm), Promega Quantifluor ST (Ex 365–395 nm, Em 440–470 nm), or Turner Biosystems TBS-380 fluorescence detector (Ex 365–395 nm, Em 440–470 nm), each set to UV mode. Samples were incubated at *in situ* temperature (PA samples; *in situ* temperatures ranged from 21.4 to 25.6°C or at room temperature (20–21°C; TN samples). For every sample, a calibration curve was made using AMC standard dissolved in MilliQ-H_2_O mixed with 860 μL sample, 100 μL phosphate buffer, and an addition of MilliQ-H_2_O to bring the total volume to 1000 μL.

pH dependence for Gly-AMC and Leu-AMC hydrolysis was measured at Ardena Brook and Belmar Inlet in 2016. For pH optimum measurements, the procedure was the same, but the buffer was phosphate-citrate universal buffer, and the pH was manipulated from 5.0 to 9.0. For these measurements, a standard curve was created at each pH, and each sample was calibrated with the corresponding calibration curve.

### Data Analysis and Quality Control

Enzyme activities were calculated using R. All data and scripts are included as supplemental data, and deposited at http://github.com/adsteen/PEEC_MXSHS. Data were manually checked for linearity, and obvious outlier fluorescence data points were removed from the dataset based on the observation that our fluorescence detectors sometimes exhibit shot noise. Samples with outlier *v*_o_ values were not removed. *V*_max_ and *K*_m_ were calculated using the non-linear least-squares fitting algorithm implemented by the nls() function in base R. Fits for which estimated *V*_max_ and *K*_m_ were both greater than 0, and for which the standard error of estimated *K*_m_ was less than the estimated value of *K*_m_, were considered valid. As a second quality control step, fits meeting those criteria but which qualitatively did not appear to fit the data well were omitted from analysis. Note that measured *K*_m_s are effective *K*_m_s, since multiple enzymes almost certainly hydrolyzed each substrate.

## Results

### Potential Kinetics of Extracellular Peptidases

**FIGURE 2:**
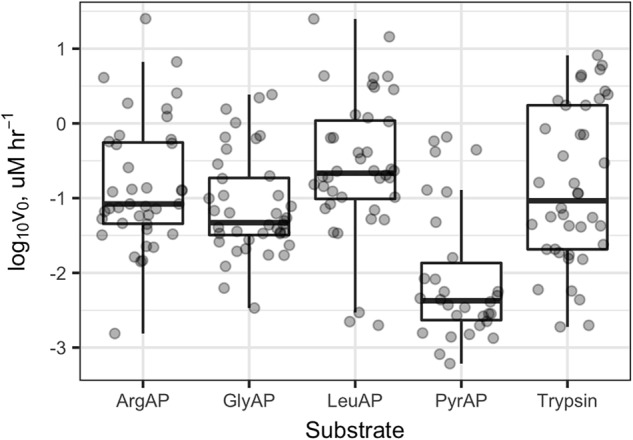
*V*_max_ values for each enzyme and sample. ArgAP, GlyAP, LeuAP, and PyrAP refer to argingine aminopeptidase, glycine aminopeptidase, leucine aminopeptidase, and pyroglutamic aminopeptidase, respectively. Horizontal lines in the boxplot boxes indicate medians and 25^th^ and 75^th^ percentiles. Vertical whiskers extend to the most extreme data point that is no further than 1.5× the interquartile range from the 25^th^ or 75^th^ percentile.

Collectively, potential enzyme activities were distributed approximately log-normally, with a geometric mean *V*_max_ of 91 nM h^-1^, a median of 73 nM h^-1^, and an interquartile range from 21 to 520 nM h^-1^ (**Supplementary Figure S1**). In Knoxville, activities were highest in Douglas Lake (DL), the TN River at VL, and a small outdoor, constructed goldfish pond (EBF). DL showed high activity both times that it was sampled. The highest activities of LeuAP were observed in the EBF and at VL. At PEEC, the highest activity was measured at sites BB1, BB2, and BS (two approximately 30-m diameter, shallow catchment ponds, and a highly turbid wetland, respectively) which were characterized by high potential ArgAP and LeuAP activities. Trypsin-like activities were consistently high in DL and at the Hesler Biology Plant Pond. One-way repeated-measures ANOVA of log-transformed *V*_max_ (*n* = 188) revealed significant differences in *V*_max_ among substrates (*p* < 0.001). Pairwise paired *t*-tests of difference in *V*_max_ means among samples revealed statistically significant differences in *V*_max_ among each pair of substrates except ArgAP and trypsin-like enzymes, which were indistinguishable (*p* > 0.05; *p*-values corrected for multiple comparisons by the Bonferroni–Holm algorithm). *V*_max_ of LeuAP was greatest, followed by ArgAP and trypsin-like enzymes, and then by GlyAP, and finally PyrAP (**Figure 2**).

**FIGURE 3:**
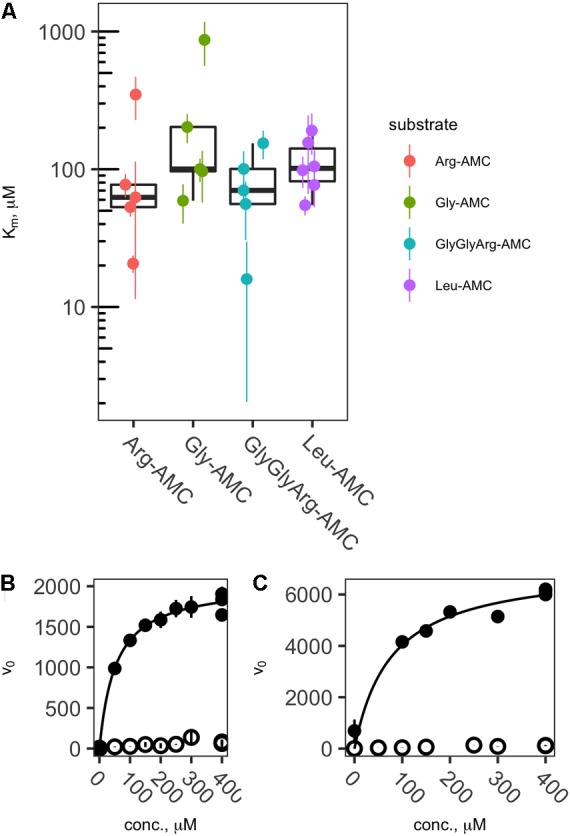
Effective *K*_m_s (**A**) and representative saturation curves **(B,C)** showing the relationship between substrate concentration and hydrolysis rate. Abbreviations are as in **Figure 2**. Saturation curves are for Arg-AMC at site DR **(B)** and Leu-AMC at site LL **(C)**.

Of the 50 sample/substrate combinations for which saturation curves were created in 2013, 20 were able to be fit to the Michaelis–Menten function, $v_{o}=\frac{V_{\max}[S]}{K_{m}+[S]}$ , where *v*_o_ is the observed rate of reaction, [*S*] is the substrate concentration, *V*_max_ is the theoretical maximum rate of reaction at infinite substrate concentration, and *K*_m_ is the effective half-saturation constant. In general, samples for which v_0_ in live samples was considerably greater than boiled controls yielded valid Michaelis–Menten fits, whereas those in which v_0_ was low did not. Thus, effective *K*_m_ values could be estimated for each peptidase except Pyr-AP, which exhibited consistently low *v*_0_. Effective *K*_m_ values ranged from a minimum of 15.6 μM to a maximum of 869 μM with a median of 101 μM and interquartile range from 66.3 to 273 μM (**Figure 3**).

**FIGURE 4:**
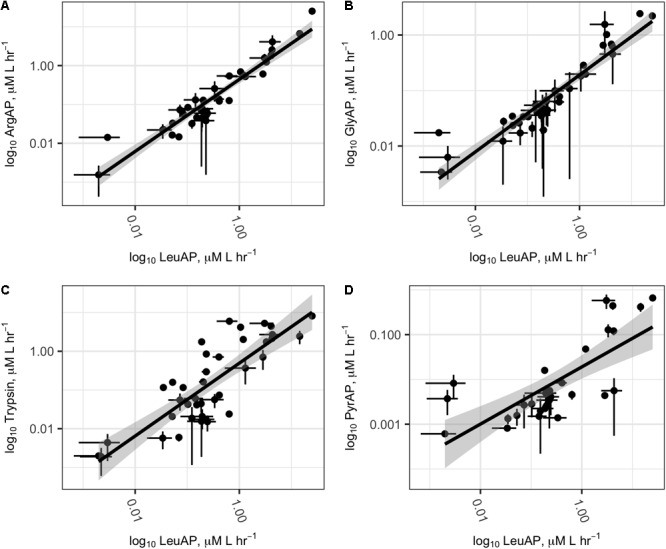
Log–log plots of potential ArgAP **(A)**, GlyAP **(B)**, Trypsin **(C)**, and PyrAP **(D)** activity as a function of potential LeuAP activity at all sites. Abbreviations are as in **Figure 2**. Dark lines represent linear regression of log-transformed potential activities, and shaded areas represent the standard error. Error bars on individual points represent the standard deviation of three replicate measurements from each site.

All potential peptidase activities were significantly intercorrelated after log transformation (**Figure 4**; ArgAP-LeuAP: slope = 0.93 ± 0.05, *n* = 39, *r*^2^= 0.90, *p* < < 0.01; GlyAP-LeuAP: slope = 0.69 ± 0.04, *n* = 40, *r*^2^ = 0.87, *p* < < 0.01; trypsin-like enzyme-LeuAP: 0.94 ± 0.10, *n* = 40, *r*^2^ = 0.68, *p* < < 0.01; PyrAP-LeuAP: slope = 0.63 ± 0.11, *r*^2^ = 0.54, *n* = 30). At individual sites, ratios of potential trypsin-like enzymes:LeuAP ranged from 0.037 to 9.3. These ratios were roughly log-normally distributed with a geometric mean of 0.53, a median of 0.46, and an interquartile range from 0.18 to 1.4 (**Supplementary Figure S2**). GlyAP and LeuAP pH dependences were indistinguishable at each site, although they were different among sites (**Supplementary Figure S3**). At Ardena Brook, both aminopeptidases were most active at pH 7.5, while at Belmar Inlet, both aminopeptidases were most active at or above pH 8.5. Potential peptidase activities were not significantly correlated to *in situ* temperature, probably because cell density or other ecological factors exerted stronger control over enzyme activity than temperature in the relatively narrow temperature range (18–32°C) sampled here.

### Intertemporal Stability of Peptidase Activity Ratios

**FIGURE 5:**
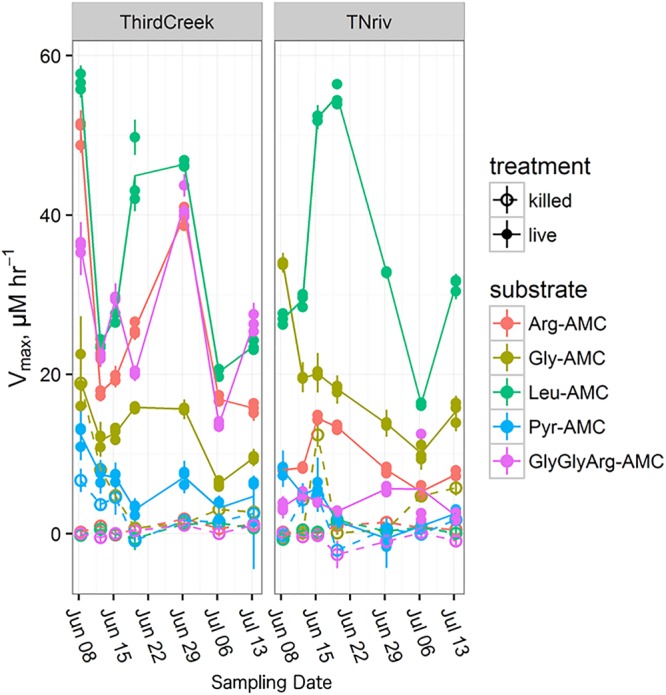
Time series over the course of a month and a half following the trends in *V*_max_ for each measured substrate at the Third Creek (ThirdCreek) and the Tennessee River at Volunteer Landing (TNriv) sampling sites. Abbreviations are as in **Figure 2**. Open circles (“killed”) represent the boiled control. Error bars represent the standard error of the estimation of *V*_max_ for each replicate. Lines are plotted through the mean of the three replicates.

Time-series measurements from Third Creek (an urban, anthropogenically impacted creek in Knoxville, TN, United States; Im et al., 2014) and in the TN River at VL (upstream of most of Knoxville’s drainage basin) indicated that patterns of enzyme activity were relatively stable on a timescale of weeks (**Figure 5**). Third Creek consistently displayed higher activity of ArgAP and trypsin-like enzymes than the TN River, which displayed higher activities of LeuAP and GlyAP. PyrAP was always negligible (but sometimes detectable) at both sites. Sites BB1 and BB2 were also sampled over multiple years and consistently showed higher LeuAP than trypsin-like potential activity.

## Discussion

The shape of the saturation curves and the fact that substrate hydrolysis rates in untreated samples were generally substantially higher than those in boiled samples indicate that the substrate hydrolysis observed here reflects activities of enzymes rather than abiotic processes. The median *K*_m_ value here, 101 μM, is somewhat higher than the median hydrolase *K*_m_ reported in a meta-analysis of extracellular enzyme kinetics, suggesting a moderately high concentration of enzyme-labile proteinaceous organic matter in the systems assayed here (Sinsabaugh et al., 2014). Potential peptidase activities (*V*_max_) in this study varied over four orders of magnitude among environments and were all mutually inter-correlated. *V*_max_ values were not significantly correlated to *in situ* temperature, likely because ecological factors (e.g., cell density and organic matter concentration) were more important than the kinetic effect of temperature in driving enzyme activity, and because the range of temperatures sampled (18–32°C) was relatively narrow. Those correlations could indicate that the assays used here report activities of two distinct enzymes, expression of which is correlated at the community level. Alternately, correlations between two substrate hydrolysis rates could indicate that the same enzyme or set of enzymes hydrolyzes multiple fluorogenic substrates. Both factors likely led to the observed data. Extracellular aminopeptidases in freshwater are relatively promiscuous, and multiple classes of aminopeptidase can hydrolyze the same substrates (Steen et al., 2015). In that study, ArgAPs were responsible for more hydrolysis of LeuAMC than were Leu-APs. The tight inter-correlation between hydrolysis rates of LeuAMC, ArgAMC, and GlyAMC, combined with the evidence for promiscuity among aminopeptidases, suggests that those substrates may have been hydrolyzed by the same enzyme or set of enzymes. This is further supported by the fact that pH dependence of GlyAP and LeuAP, which were indistinguishable from each other at two different sites, despite varying among sites (**Supplementary Figure S3**). The fact that Leu-AMC was consistently the fastest hydrolyzed substrate suggests that LeuAP, rather than GlyAP or ArgAP, was responsible for most of that hydrolysis.

The correlations between Leu-AMC and Z-GlyGlyArg-AMC hydrolysis rates and between Leu-AMC and Pyr-AMC hydrolysis rates (*r*^2^ = 0.68 and 0.63, respectively) were considerably looser than the correlations between Leu-AMC and Arg-AMC or Gly-AMC hydrolysis rates (*r*^2^ = 0.90 and 0.87, respectively). Correspondingly, the ratios of Z-GlyGlyArg-AMC and Pyr-AMC to Leu-AMC hydrolysis rates at individual sites were significantly more variable than ratios of Arg-AMC and Gly-AMC to Leu-AMC hydrolysis rates (**Figure 4** and **Supplementary Figure S2**). These facts suggest that, while Leu-AMC, Gly-AMC, and Arg-AMC were likely hydrolyzed by the same set of enzymes, different sets of enzymes hydrolyzed Z-GlyGlyArg-AMC and Pyr-AMC. This makes sense from a biochemical perspective: the unusual cyclic lactam structure of pyroglutamc acid is a poor fit for the active site of a typical aminopeptidase, and indeed *N*-terminal pyroglutamic acid acts to protect peptides from intracellular hydrolysis by aminopeptidases (Kumar and Bachhawat, 2012). Aminopeptidases specific for pyroglutamic acid have been identified (EC 3.4.19.3, Awadé et al., 1994), and pyroglutamic acid is a minor component of some proteins relevant to aquatic systems, such as bacteriorhodopsin (Blanck et al., 1989). Thus, it is plausible that the hydrolysis of Pyr-AMC observed in these samples was due to pyroglutamic aminopeptidase, but given the low activities observed, we cannot exclude the possibility that Pyr-AMC hydrolysis was primarily due to some other set of peptidases, possibly including peptidases that were not directly assayed here.

Z-GlyGlyArgAMC is a nominally a substrate for trypsin, a broad-spectrum endopeptidase (i.e., peptidase that hydrolyzes proteins from the middle rather than the ends) (Obayashi and Suzuki, 2005). The bulky Z- group effectively prevents hydrolysis by aminopeptidases (Nakadai and Nasuno, 1977), and given the broad range of observed ratios of Z-GlyGlyArg-AMC:Leu-AMC hydrolysis rates, it is likely that that Z-GlyGlyArg-AMC and the single-amino acid substrates were hydrolyzed by distinct enzymes. Thus, the broad correlation in hydrolysis rates between those two substrates is probably due to community-level co-expression of trypsin-like enzymes and the common set of aminopeptidases that hydrolyzed Leu-AMC, Arg-AMC, and Gly-AMC.

It has long been recognized that a variety of peptidases are potentially present in aquatic environments (Christian and Karl, 1998). Early evidence suggested that assays of a single peptidase substrate provide a reasonable approximation of the total peptidolytic potential of a microbial community, because extracellular peptidases are frequently capable of acting on a wide range of peptides (Nomoto et al., 1960). The promiscuity of aquatic peptidases was used as justification for fluorogenic substrate-based enzyme assays when that technique was first adopted for aquatic samples (Hoppe, 1983), and indeed it appears that Leu-AMC hydrolysis is caused by a range of aminopeptidases in aquatic environments (Steen et al., 2015).

The results presented here place further constraint on the degree to which measurement of the hydrolysis rate of a single substrate is a useful measure of the total peptidolytic capacity of an ecosystem. LeuAP potential activity does correlate well with the activity of other aminopeptidases across a broad range of systems. For studies that examine systems in which activity varies by several orders of magnitude (for instance, studies that use LeuAP as a proxy for N demand across diverse environments, e.g., Sinsabaugh et al., 2009), LeuAP activity correlates well enough with endopeptidase activity that the additional information, time, and expense required to assay multiple peptidases are not justified given the novel information those measurements yield. In studies that have a narrower domain, for instance, time-series analyses in which LeuAP activity might vary within an order of magnitude (Allison et al., 2012; Mahmoudi et al., 2017) – this assumption is more dangerous, as changes in the ratio of endopeptidases : aminopeptidases could obscure patterns observed in just one peptidase. In this study, the ratio of trypsin-like potential activity to LeuAP potential activities ranged from 0.037 to 9.3. If the sum of trypsin-like activity and LeuAP activity places a lower bound on the total peptidolytic capacity of a system, then LeuAP could represent anywhere from 9.7 to 96% of total peptidolytic capacity, representing about an order of magnitude of potential error. Furthermore, since the endopeptidase:aminopeptidase activity appears to be a non-stochastic feature of ecosystems, this error would be systematic rather than random. Studies in which the range of LeuAP activities is narrower than an order of magnitude or so, assaying a broader set of peptidases, including endopeptidases and aminopeptidases, may yield a more complete picture of the potential for protein degradation.

Heterotrophic processes in aquatic systems are often described in chemically non-specific terms, such as “N acquisition” or “protein degradation.” This is a useful way to distill important ecological patterns from the tremendously complex set of biochemical pathways that may be active in a system. It also flows from the limitations of organic geochemistry analytical technology: at present, it is relatively straightforward to measure the concentration of “hydrolysable amino acids” (i.e., protein-like material) in aquatic systems, but very challenging to measure concentrations of specific proteins (Moore et al., 2012). Microorganisms sense and interact with the world at much finer chemical resolution. Those fine-scale interactions with the environment are reflected in the expression of specific extracellular enzymes. Measuring a broader set of extracellular enzymes can therefore yield insight into how microorganisms interact with their chemical environment. These results indicate that Leu-AMC hydrolysis is an acceptable proxy for total peptidolytic capacity of an environment only when the potential LeuAP activities vary over several orders of magnitude. When potential LeuAP activities span about an order of magnitude or less, variability in the aminopeptidase:endopeptidase ratio may cause total peptidolytic capacity to become decoupled from potential LeuAP activity. In such a data set, assays for multiple peptidases should be included to capture variability in total community peptidolytic potential.

## Members of the Malcolm X Shabazz High School Aquatic Biogeochemistry Team

Ivy Adu-Poku, Saskieya Anderson, Kiara Amore, Tequan Anderson, Elmy Antonio, Delvon Artis, Monai Barnes, Manusha Bearfield, Kim Boerrigter, Antwon Bowman, Zion Brummel, Abdul Bryant, Tyre Bush, Clervens Clerjuste, Latonya Coates, Ameena Corney, Jamie Crosby, Abdul Crowley, Michelle Culver, Leon Cummings, Zyion Eastmond, Genaro Falcon, Ciara Gilette, Lexus Gonzalez, Brianna Grant, Dequan Graves, Christian Hardy, Jamar Harris, Kevin Harrison, Deshawn Hart, Ismail Hinds, Latifa Hinds, Samantha Hunter, Imade Igiebor, Teniyjaa Jacobs, Malachi Jefferson, Shani Ketema, Zack Ketema, Ahmad Lancaster, Camille Laurel, Ferard Majette, Imani McCray, Francis Mensa, Elizabeth Mensah, Justin Mestre, Tai-Xian Mitchell, Davon Moody, Ivana Negron, Zaire O‘Neill, Okoye Onyebuchi, Nasir Parham-Sanders, Madelyn Perez, Chris Pitt, Briana Racine, Acestra Robinson-Williams, Elvis Sanchez, Jabryant Vines, Jacelyn Valenciano, Nadira Wilkins, Anzunai Williams, Ma-Lisa Winborne, Henasia Wilson, and Yasmiah Wilson.

## Author Contributions

LM and ABB helped design the sampling plan, performed experiments, analyzed the data, and helped write the manuscript. MXSHS-ABT (a group author) and NF performed experiments and analyzed the data. KB designed and performed experiments and analyzed the data. JR helped design the sampling plan. PM initiated the project, helped design the sampling plan, and performed experiments. AS initiated the project, helped design the sampling plan, performed experiments, analyzed the data, and helped write the manuscript.

## Conflict of Interest Statement

The authors declare that the research was conducted in the absence of any commercial or financial relationships that could be construed as a potential conflict of interest.

## Supplementary Material

FIGURE S1 Density plot of all Vmax values measured in this study, showing the approximate log-normal distribution of activities.

FIGURE S2 Distribution of ratios of trypsin-like potential activity to LeuAP potential activity, showing the approximate log-normal distribution of activities.

FIGURE S3 pH dependence of LeuAP (marked LeuAMC) and GlyAP (marked GlyAMC) at Ardena Brook (ARD) and Belmar Inlet (BLMR).
