# Supplementary figures and images for: Potential Activities of Freshwater Exo- and Endo-Acting Extracellular Peptidases in East Tennessee and the Pocono Mountains
Source: Front Microbiol. 2018 Mar 6;9:368. doi: 10.3389/fmicb.2018.00368 (PMC5845674; doi:10.3389/fmicb.2018.00368)

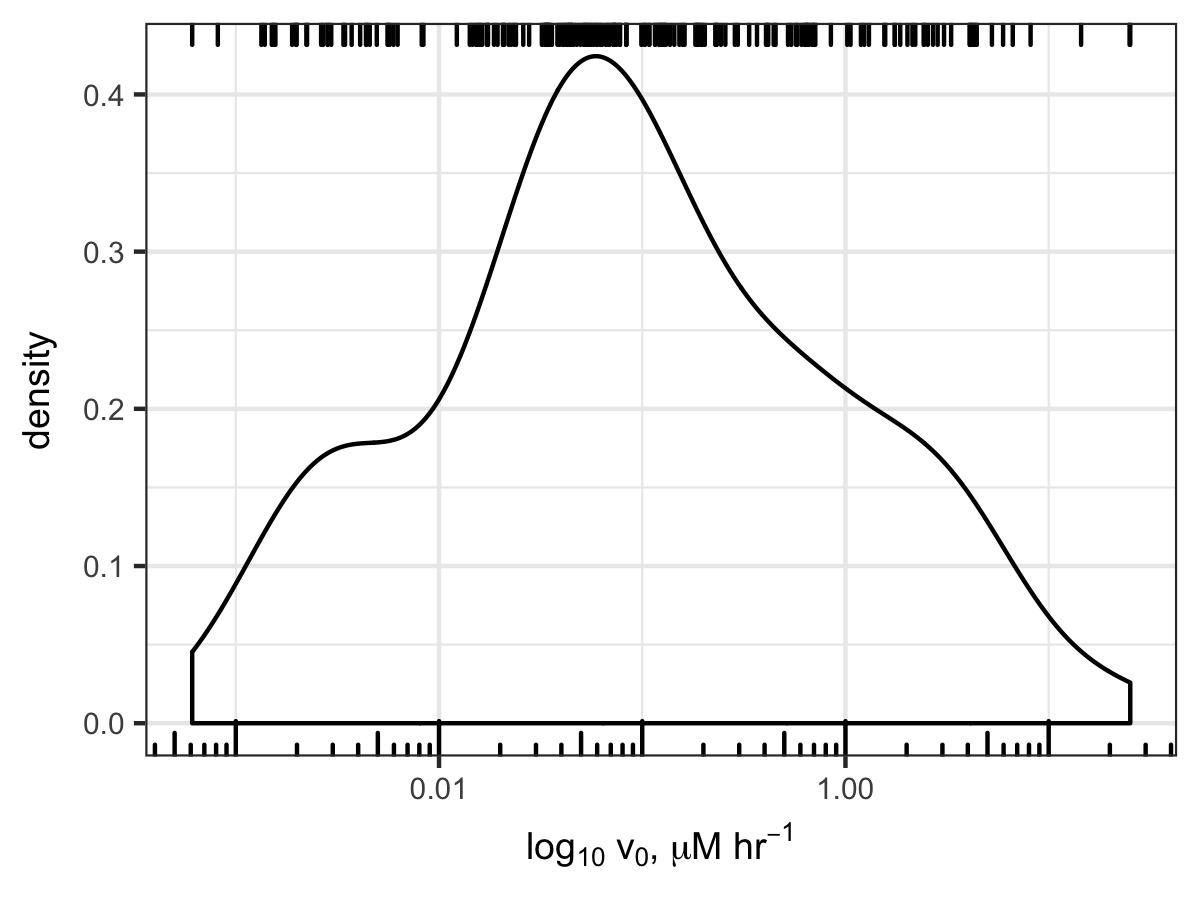

Supplement: FIGURE S1 — Density plot of all Vmax values measured in this study, showing the approximate log-normal distribution of activities. [file Image_1.png]

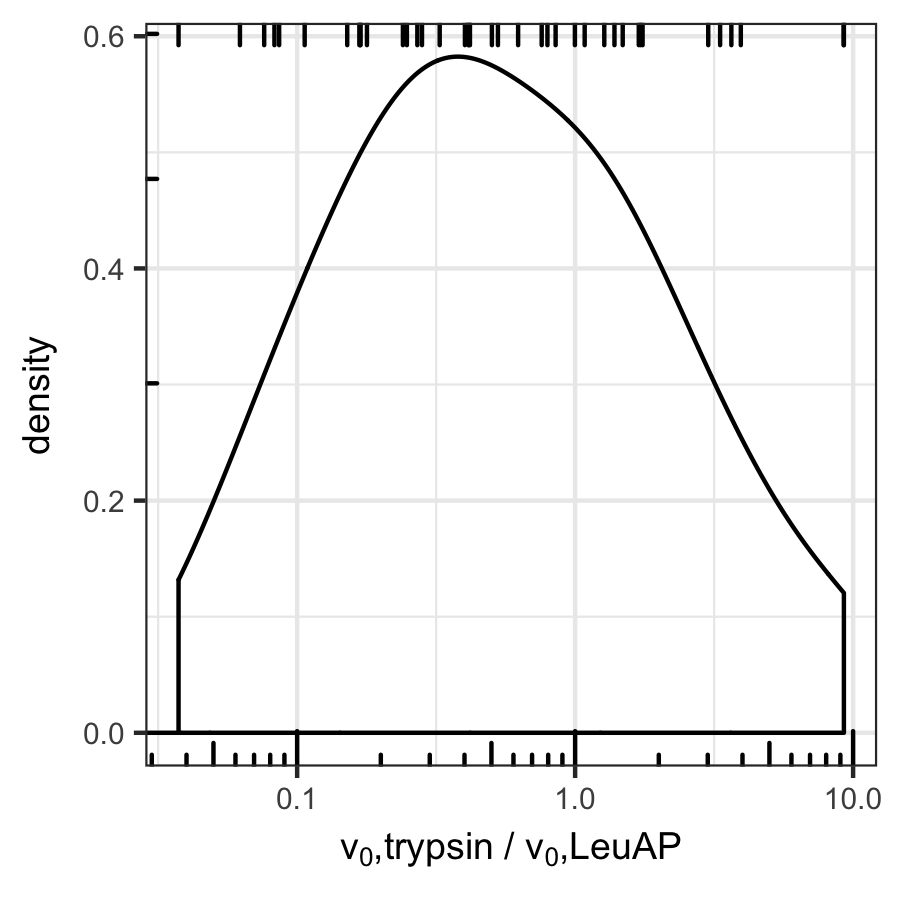

Supplement: FIGURE S2 — Distribution of ratios of trypsin-like potential activity to LeuAP potential activity, showing the approximate log-normal distribution of activities. [file Image_2.tiff]

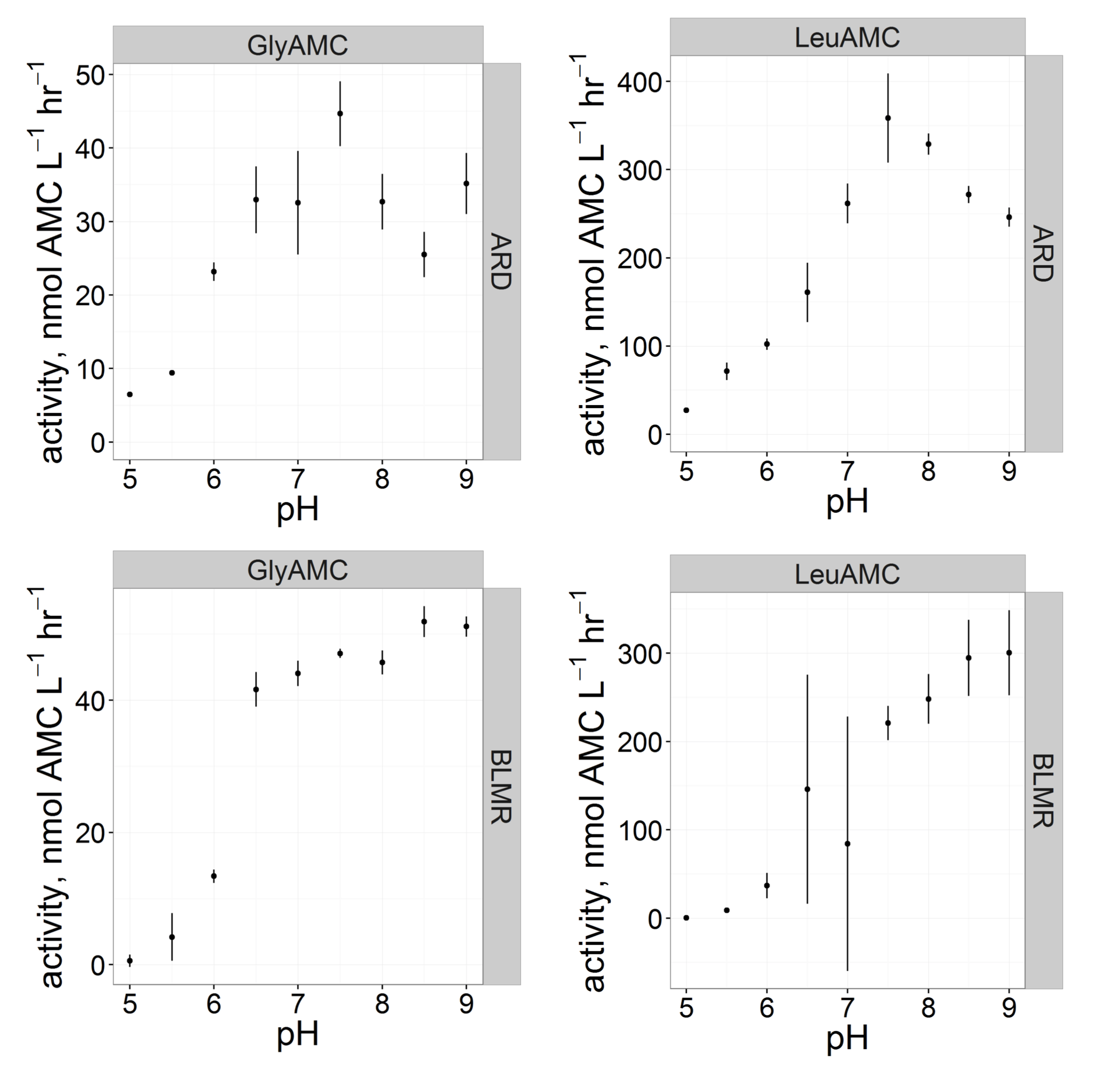

Supplement: FIGURE S3 — pH dependence of LeuAP (marked LeuAMC) and GlyAP (marked GlyAMC) at Ardena Brook (ARD) and Belmar Inlet (BLMR). [file Image_3.png]
